# Disease-associated adipose browning: current evidence and perspectives

**DOI:** 10.1080/21623945.2025.2610540

**Published:** 2026-01-07

**Authors:** Xiyue Zhang, Xue Han, Jiesi Xu, Guoping Li

**Affiliations:** The Key Laboratory of Geriatrics, Beijing Institute of Geriatrics, Institute of Geriatric Medicine, Chinese Academy of Medical Sciences, Beijing Hospital/National Center of Gerontology of National Health Commission, Beijing, P.R. China

**Keywords:** Disease-associated adipose browning, cancer, kidney health, burn injury, atherosclerosis

## Abstract

Brown and beige adipose tissue represent evolutionary adaptations in mammals, functioning as specialized thermogenic organs to maintain body temperature. Over the past two decades, researches have demonstrated that white adipose tissue (WAT) browning is an effective strategy to enhance energy expenditure. However, a growing body of evidence indicates that the browning process frequently occurs in a variety of chronic disease states, though its pathophysiological significance remains unclear. This review summarized evidence of pathological browning observed in human diseases and animal models, including breast cancer, colorectal cancer (CRC), clear cell renal cell carcinoma (ccRCC), kidney health, burn injury, atherosclerotic, SARS-CoV-2 and sepsis. Despite distinct pathological contexts, adipose tissue browning is consistently observed. This suggests that browning may not simply serve its classical metabolically protective role, but instead reflect an atypical response to pathological stress. It is currently unclear whether this is a compensatory mechanism by the organism in a diseased state or merely a byproduct of the disease process. Whether this response is adaptive or a cause of disease progression remains unresolved. Future research should therefore focus on identifying the triggers and functional outcomes of pathological browning to better understand adipocyte plasticity and its role in disease progression.

## 1. Introduction

Brown adipose tissue (BAT) and inducible beige adipose tissue (beige AT) residing within white adipose depots-collectively termed BAT-are key thermogenic organs that evolved in mammals as an adaptation to cold environments. They convert chemical energy into heat through uncoupling protein 1 (UCP1)-mediated non-shivering thermogenesis, protecting neonates and mammals from hypothermia [1,2].

Although BAT was initially discovered as early as the 1950s, it was not until the early 21st century, with the identification of metabolically active BAT in adults [3], that a new era of research commenced. This era focused on harnessing BAT to increase energy expenditure and combat metabolic diseases. Over the past two decades, driven by the global epidemic of chronic metabolic disorders like obesity, mainstream scientific research has focused almost exclusively on the beneficial metabolic roles of BAT. These include enhancing energy expenditure, promoting glucose/lipid clearance, improving insulin sensitivity, and countering metabolic diseases [4–6].

However, this therapeutic optimism may overlook a fundamental biological truth: physiological systems evolved for survival in ancestral environments may incur unintended adverse consequences in our modern context of energy abundance. Crucially, could the excessive or sustained activation of BAT thermogenesis become detrimental under specific physiological or pathological conditions?

While the metabolic benefits of adipose tissue browning have been extensively reviewed, a systematic analysis of its potential adverse consequences remains strikingly absent. A growing body of evidence indicates that the phenomenon of browning occurs in WAT under various pathological conditions. This review cautions against overstating the benefits of browning and emphasizes its impact on adipose metabolic homoeostasis, particularly in specific pathophysiological contexts.

## 2. Literature search and review rationale

Defined as a narrative review, this study integrates findings from human clinical studies and preclinical animal models to ensure cross-context validity. A structured literature search was conducted across PubMed, Web of Science, and Scopus databases up to [Month, Year] using the following Boolean keyword combinations: (‘adipose browning’ OR ‘beige adipocyte’ OR ‘WAT browning’) AND (‘breast cancer’ OR ‘kidney disease’ OR ‘burn injury’ OR ‘atherosclerosis’ OR ‘SARS-CoV-2’ OR ‘pathological stress’). Included works were peer-reviewed English publications (original research articles, focused reviews) that explicitly investigate adipose browning in the aforementioned pathological contexts; Excluded were non-English articles, abstract-only studies, case reports, and research unrelated to the core theme of disease-associated browning. This targeted strategy ensured a focused synthesis of browning’s pathological roles, laying the groundwork to clarify its functional duality and inform future mechanistic and translational research.

## 3. Pathological adipose browning in cancer

Adipose tissue browning, once recognized as a beneficial metabolic adaptation, emerges as a pathological driver in multiple cancer types-including breast cancer, CRC and ccRCC. Across these malignancies, tumour-induced abnormal browning of adjacent adipose tissue remodels metabolic crosstalk between adipocytes and cancer cells, ultimately promoting disease progression, cachexia, and poor patient outcomes.

### 3.1. Pathological adipose browning in breast cancer

Breast cancer is one of the most prevalent malignancies worldwide, representing a significant public health challenge [7,8]. Emerging evidence indicates that breast cancer -associated adipocytes play a critical role in the tumour microenvironment, influencing the progression of the disease [9].

The presence of UCP1-positive adipocytes in mammary adipose tissue has been established [10,11]. Gene expression analyses further reveal elevated levels of classic brown (MYF5, EVA1, OPLAH) and beige (CD137/TNFRSF9, TBX1) adipocyte markers, along with PRDM16-a key regulator of brown adipocyte differentiation-in breast cancer xenografts [12–14]. Moreover, the adipose tissue surrounding malignant tumours undergoes a greater degree of browning compared to that near normal breast tissue [14]. Advanced-stage breast cancer patients are particularly susceptible to weight loss and cancer-related cachexia, conditions exacerbated by the enhanced browning of adipose tissue [15]. Conversely, depletion of UCP1-positive cells markedly suppresses tumour development [14]. Cancer-associated cachexia is a multifactorial syndrome caused by pro-inflammatory factors, metabolic factors and other substances secreted by tumours and their microenvironment, which is characterized by the progressive loss of skeletal muscle and adipose tissue. Excessive catabolism and energy imbalance increase the mortality risk of cancer patients [16,17].

Multiple factors contribute to adipocyte browning in breast cancer, including molecules like exosomes and adrenomedullin secreted by breast tumour cells and metabolic changes in the breast tumour microenvironment.

#### 3.1.1. Breast tumours derived extracellular vesicles stimulates browning

Small extracellular vesicles (sEVs), especially exosomes, have been recognized as critical mediators of intercellular communication, connecting primary tumours with distant metabolic organs and exerting systemic effects on disease progression [18,19]. WAT browning has been strongly associated with cancer-derived exosomes [20]. For instance, S. Sun et al. showed that exosomes from breast cancer promote WAT browning via delivery of miR-155, which downregulates UBQLN1 expression [21], which encodes a ubiquitination-related protein known to be involved in cancer progression [22]. Conversely, overexpression of UBQLN1 was found to reverse exosome-induced adipose browning in mouse models of breast cancer cachexia [21].

Additionally, emerging evidence indicates that breast cancer-derived exosomes modulate leptin signalling pathways, further promoting WAT browning [21]. Leptin, a hormone produced mainly by adipose tissue, plays a central role in regulating energy balance and body weight [23,24]. It stimulates lipolysis and thermogenesis by triggering catecholamine release from neurons, leading to activation of the NE-β3 adrenergic receptor (β3-AR) and protein kinase A (PKA) signalling [25]. Elevated serum leptin levels are frequently observed in breast cancer patients, a phenomenon closely linked to obesity-since adipose tissue is the primary source of leptin, excess adiposity (especially visceral obesity) drives chronic hyperleptinaemia [26]. This obesity-associated hyperleptinaemia is not merely a metabolic byproduct but also strengthens the connection between leptin and breast cancer advancement: obese breast cancer patients exhibit higher leptin levels than non-obese counterparts, and this elevation correlates with larger tumour size, increased lymph node metastasis, and poorer prognosis [27,28]. Beyond its metabolic roles, leptin also promotes angiogenesis and endothelial cell migration within the tumour microenvironment [29,30]. By binding leptin receptors on breast cancer cells, it induces vascular endothelial growth factor (VEGF) production, thereby enhancing angiogenic signalling and supporting tumour growth [31].

Moreover, Yong Hu et al. revealed that exosomal miR-204-5p upregulates hypoxia-inducible factor 1A (HIF1α) in WAT by targeting the von Hippel-Lindau (VHL) tumour suppressor gene [32,33]. This induction of HIF1A activates leptin signalling and stimulates lipolysis, contributing to adipose browning. Notably, adenovirus-mediated overexpression of VHL attenuated miR-204-5p-induced lipolysis and browning, suggesting a protective strategy against cancer-associated cachexia. Here, lipolysis refers to the process where the activity of lipolysis-related enzymes is increased in adipose tissue, accompanied by elevated free fatty acids in the serum and reduced triglyceride levels [34].

Together, these findings demonstrate the role of tumour-derived exosomes in regulating WAT browning through multiple molecular mechanisms.

#### 3.1.2. Breast tumours secreted adrenomedullin stimulates browning

Breast tumours secrete a variety of factors that modify the phenotype and function of adjacent adipocytes, among which adrenomedullin (ADM) has been identified as a key mediator [35]. Produced within breast cancer mammospheres, ADM exhibits pleiotropic biological effects, including vasodilation and the regulation of cell proliferation, apoptosis, and inflammation [36]. Recent evidence indicates that ADM can stimulate lipolysis and promote adipocyte browning. Under hypoxic conditions, adipose tissue upregulates ADM production, which in turn enhances UCP1 expression and hormone-sensitive lipase (HSL) phosphorylation, thereby triggering lipolysis and facilitating the formation of beige adipocytes [35]. This lipolytic process supplies free fatty acids (FFAs) that serve as a critical energy source for tumour growth [37].

Beyond its effects on breast epithelial cells, ADM contributes to tumour progression by influencing endothelial cell behaviours-such as proliferation, migration, and apoptosis-thereby remodelling the tumour microenvironment [35,38]. Additionally, ADM has been shown to impair anti-tumour immunity by suppressing cytokine secretion and dampening immune surveillance [35]. These findings collectively support a model of a vicious cycle: breast cancer-derived ADM drives both tumour expansion and metabolic reprogramming of adipose tissue, which in turn further sustains cancer progression. Nonetheless, the detailed mechanisms by which ADM modulates the tumour microenvironment and aggravates malignancy remain incompletely elucidated. Further research is needed to decipher these pathways, which may reveal novel therapeutic opportunities to disrupt this cycle and improve patient outcomes. In pathological contexts, lipolysis supplies the vital ‘fuel’ for the thermogenic function of beige adipocytes, while the signalling pathways mediating browning reciprocally enhance lipolysis-together forming a highly efficient energy-consuming cycle.

**Figure 1.**
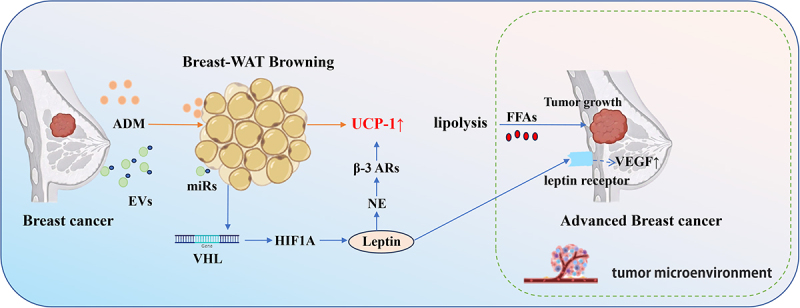
Breast cancer cell-derived ADM and EVs promote the browning of mammary WAT.

Current research linking breast cancer-associated adipocyte browning (elevated UCP1/thermogenic markers) to tumour progression and cachexia is limited by over-reliance on preclinical models, neglected subtype-specific differences, and unclear crosstalk between exosomes/ADM (Figure 1). Therapeutically, targeting these pro-browning mediators (e.g. miR-155, ADM) or repurposing metabolic drugs (β3-AR antagonists, PPARγ agonists), with UCP1/PRDM16 as prognostic markers, offers actionable strategies for advanced patients, especially those with cachexia.

Breast cancer cell-derived adrenomedullin (ADM) enhances the expression of UCP1 in adipocytes and promotes the phosphorylation of hormone-sensitive lipase (HSL), thereby stimulating lipolysis and the browning of adipocytes. Breast cancer cell-derived extracellular vehicles (EVs) deliver miRNA to target the VHL gene, leading to the upregulation of hypoxia-inducing factor 1A (HIF1A) in WAT. This activates the leptin signalling pathway and triggers WAT browning and adipose atrophy. The browning of breast-WAT is accompanied by enhanced fatty acid mobilization, with released free fatty acids serving as an energy supply for tumour growth. Additionally, elevated serum leptin levels in breast cancer patients can directly interact with tumour stroma, activating leptin receptors on cancer cells to induce VEGF production.

The Schematic model was created using BioRender (https://www.biorender.com/) and Adobe Illustrator.

### 3.2. Pathological adipose browning in colorectal cancer

In the progression of CRC, adipose tissue browning emerges as a critical pathological feature associated with disease deterioration. It is directly correlated with the development of cancer cachexia, enhanced tumour invasiveness and metastatic potential, as well as poor patient prognosis [39,40].

Two major molecular mechanisms underlying adipose tissue browning-driven CRC progression have been identified:

#### 3.2.1. Colorectal cancer-derived exosomal miR-146b-5p stimulates browning

**Figure 2.**
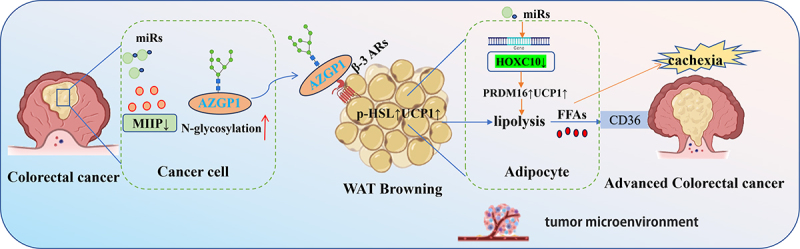
Colorectal cancer cell-derived exosomal and downregulated MIIP induce the browning of mammary WAT.

CRC cells secrete exosomes enriched in miR-146b-5p. Upon internalization by white adipocytes, this microRNA specifically inhibits the expression of its downstream target gene HOXC10, disrupting the normal metabolic homoeostasis of adipocytes and inducing WAT browning. Browning accelerates lipolysis: on one hand, this causes excessive systemic energy dissipation, triggering cancer cachexia characterized by abrupt weight loss and generalized debility. On the other hand, the released metabolic byproducts provide essential energy support for cancer cell proliferation, further fuelling tumour progression (Figure 2). This mechanism was validated in a comparative study involving tissue samples from 48 CRC patients and healthy individuals [39].

#### 3.2.2. Colorectal cancer-downregulated MIIP stimulates browning

The expression of migration and invasion inhibitory protein (MIIP) is significantly reduced in CRC tissues, and MIIP levels exhibit a negative correlation with the degree of peritumoral adipose tissue browning. MIIP downregulation stimulates tumour cells to secrete glycosylated AZGP1 protein, which acts on adipocytes to activate the intracellular cyclic adenosine monophosphate (cAMP)-PKA signalling pathway-subsequently inducing adipocyte browning and lipolysis (Figure 2). The released free fatty acids (FFAs) are taken up by CRC cells, not only providing sufficient energy for cancer cell proliferation and invasion but also enhancing their anti-apoptotic capacity, thereby accelerating local tumour infiltration and distant metastasis. Additionally, drug intervention experiments have confirmed that inducing adipose tissue browning can disrupt tumour energy supply, inversely validating the core role of this metabolic crosstalk mechanism in CRC progression [40].

Translating these findings to therapy, we validate that combining chemotherapy with WAT browning inhibitors (targeting CRC-adipose metabolic crosstalk) or FFA uptake blockers (cutting off tumour energy supply) boosts anti-cancer efficacy-establishing a mechanism-based framework for CRC patients with aberrant MIIP expression.

Current CRC-adipose browning research is limited by small clinical cohorts, insufficient study of subtype differences and unclear crosstalk between the miR-146b-5p/HOXC10 and MIIP/AZGP1 pathways, while targeting pro-browning mechanisms paired with browning-related biomarkers for patient stratification could improve outcomes in advanced, especially cachectic, CRC patients.

Colorectal cancer cells induce WAT browning via two mechanisms: (1) Tumour-derived exosomal miR-146b-5p suppresses adipocyte HOXC10 expression, upregulating browning markers (UCP1, PRDM16) to initiate lipolysis and contribute to cancer cachexia; (2) Reduced MIIP expression in CRC cells stimulates secretion of glycosylated AZGP1, which activates the adipocyte β3-adrenergic receptor (β3-AR) signalling pathway to elevate p-HSL and UCP1, amplifying lipolytic activity. Liberated free fatty acids (FFAs) are subsequently taken up by CRC cells via CD36, sustaining the metabolic demands of advanced tumour progression.

The Schematic model was created using BioRender (https://www.biorender.com/) and Adobe Illustrator.

### 3.3. Pathological adipose browning in renal cell carcinoma

Perirenal adipose tissue (PAT) represents a unique visceral fat depot that lies between the renal fascia and renal capsule, encircling the kidney to fulfil a supportive role in its physiological functions [41]. In the course of tumour development, ccRCC cells are capable of penetrating the renal capsule and invading into thePAT-a pathological process closely linked to an unfavourable clinical prognosis [42,43]. There is a bi-directional communication between ccRCC cells and the PAT. Recent studies have confirmed a novel role for PAT browning in exacerbating renal cell carcinoma [44].

Renal cell carcinoma is a fatal and highly prevalent tumour, with ccRCC being the most common pathological type of kidney cancer [45–47].

Studies have shown that the browning of PAT adjacent to ccRCC tumours is significantly enhanced in clinical samples [48]. ccRCC can secrete parathyroid hormone-related protein (PTHrP) and thus promoting PAT browning (Figure 2). The abnormal high expression of PTHrP activates the PKA signalling pathway, thereby inducing the browning of PAT [48].

**Figure 3.**
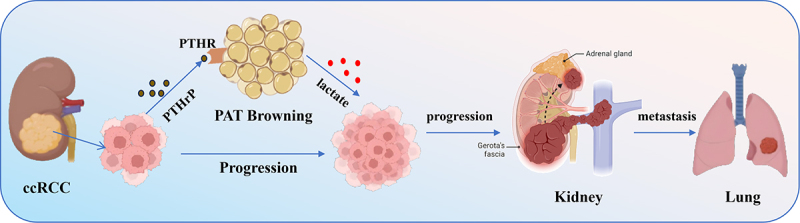
CCRCC secretes PTHrP, which stimulates the browning of PAT and accelerates tumor progression and metastasis.

It has been reported that the browned PAT enhances the glycolytic process due to thermogenic demand, releasing excessive lactic acid into the tumour microenvironment. ccRCC-secreted PTHrP induces PAT browning, which significantly boosts lactate production and release via three integrated mechanisms. First, browning-driven thermogenic demand (mediated by UCP1) triggers metabolic reprogramming, enhancing glycolysis as evidenced by elevated extracellular acidification rate (ECAR) and upregulated glycolytic enzymes like lactate dehydrogenase A (LDHA), with mitochondria prioritizing thermogenesis over oxidative metabolism. Second, UCP1-induced mitochondrial uncoupling blocks pyruvate entry into the TCA cycle; Accumulated cytoplasmic pyruvate is efficiently converted to lactate by LDHA, which regenerates NAD^+^ to sustain glycolysis. PTHrP doubles lactate secretion in a short time, while Pgc1a or Ucp1 knockout abrogates this effect. Third, browning upregulates monocarboxylate transporters 1 and 4 (MCT1/4) to facilitate efficient lactate release into the tumour microenvironment, an effect reversible by the MCT1/4 inhibitor 7ACC1 [44,49,50]. The kidneys are crucial for lactic acid metabolism. In conditions such as acute kidney injury and diabetic nephropathy, the kidneys’ ability to metabolize lactate is impaired, leading to lactate accumulation and exacerbating renal dysfunction [51]. Lactate is involved in tumour promotion and tumour progression of ccRCC [52]. There is evidence suggesting that ccRCC cells can take up lactic acid through the MCT1/4 transporter and use it as a carbon source to participate in the tricarboxylic acid cycle, thereby promoting their own proliferation, invasion and metastasis [48]. PTHrP-stimulated beige adipocytes show enhanced glycolysis and increased lactate secretion, which in turn provides fuel for ccRCC progression (Figure 3), blocking lactate efflux from these adipocytes reduces the tumorigenic potential of ccRCC [44]. In vivo experiments also confirmed that blocking the interactions related to this browning process (such as inhibiting the PKA pathway) can reduce pulmonary metastasis of ccRCC [48].

ccRCC secretes PTHrP, which binds to PTHR on adipocytes to drive perirenal adipose tissue (PAT) browning. This PAT browning triggers lactate release, which fuels ccRCC progression and promotes the tumour’s metastasis to the lung.

The Schematic model was created using BioRender (https://www.biorender.com/) and Adobe Illustrator.

Future research should expand beyond breast cancer, CRC and ccRCC to investigate adipose browning in more cancer models, with consideration of inter-individual differences related to sex and age. It is also necessary to clarify the dynamic expression patterns of adipose browning-related biomarkers and translate them into clinical indicators, so as to develop targeted therapies that disrupt tumour-adipose metabolic crosstalk, such as inhibiting pro-browning signalling or blocking nutrient supply from browned adipocytes to tumours.

## 4. Pathological adipose browning in chronic kidney disease

### 4.1. Pathological adipose browning stimulate energy wasting in uraemia

Chronic kidney disease (CKD) is one of the most prevalent organ dysfunctions, with its incidence steadily increasing over the past decade [53]. Impaired kidney function leads to the systemic accumulation of various metabolic waste compounds, collectively referred to as uraemic toxins [54]. The progressive retention and build-up of these toxins characterize uraemic syndrome, which imposes a heavy burden on patients and has adverse effects on their bodies [55]. Uraemic toxins encompass a wide range of substances, including protein metabolism byproducts (asymmetric dimethylarginine, indoxyl sulphate, p-cresyl sulphate, urea), cytokines (tumour necrosis factor α [TNF-α]), interleukins (interleukin-1β [IL-1β] and interleukin-6 [IL-6]), advanced glycosylation end products, and other metabolites (atrial natriuretic peptide), all of which are typically cleared by healthy kidneys [56,57]. Studies using adipocyte and rodent models of kidney disease have demonstrated that uraemic toxins play a significant role in modulating adipose tissue. For instance, p-cresyl sulphate has been identified as a mediator of fat atrophy, suggesting its potential involvement in the activation of brown and beige adipocytes [54,58,59].

Elevated levels of uraemic toxins in the serum of uraemic patients can induce increased expression of UCP1 and peroxisome proliferator-activated receptor gamma coactivator 1- alpha (PGC1α) in primary adipocytes. Among these uraemic toxins, natriuretic peptides are particularly notable for their role in activating browning within the uraemic environment [60,61]. Atrial natriuretic peptide, brain natriuretic peptide, and N-terminal pro-B-type natriuretic peptide (NT-proBNP) are retained in CKD and are associated with adverse clinical outcomes [62]. In CKD mouse models, elevated levels of ANP have been observed, with its accumulation potentially activating WAT browning by upregulating the expression of Ucp1 and Pgc1a. This process increases resting energy expenditure and promotes a hypermetabolic state, ultimately contributing to protein-energy wasting in CKD patients [63].

The role of browning in the pathogenesis of CKD has garnered increasing attention. Evidence suggests that browning is associated with elevated protein-energy wasting in CKD patients, which leads to poorer survival outcomes. Protein-energy wasting is characterized by weight loss and elevated resting energy expenditure, both of which are exacerbated by browning [64,65]. In haemodialysis patients, the concentration of NT-proBNP is positively correlated with the number of protein-energy wasting components and negatively correlated with albumin levels, further worsening CKD progression [66]. Atrial natriuretic peptide and brain natriuretic peptide have been shown to stimulate lipolysis in human adipocytes and the upregulate Ucp1 expression in WAT, along with increased resting energy expenditure in mice injected with BNP, suggests that BNP may be involved in to browning and PEW in CKD [63]. Elevated thermogenic activities in both BAT and WAT browning significantly contribute to resting energy expenditure in both rodents and humans, thus potentially aggravating the progression of CKD [63].

### 4.2. Adipose browning exacerbates CKD-associated cachexia through parathyroid hormone

Patients with CKD frequently develop cachexia, a complex metabolic syndrome characterized by progressive muscle atrophy, increased energy expenditure, and systemic inflammation [67]. Cachexia in the context of CKD represents a complex metabolic derangement, encompassing anorexia, progressive weight loss, atrophy of adipose tissue and skeletal muscle, and an elevated metabolic rate [68,69]. Caloric supplementation has proven ineffective in reversing these effects.

**Figure 4.**
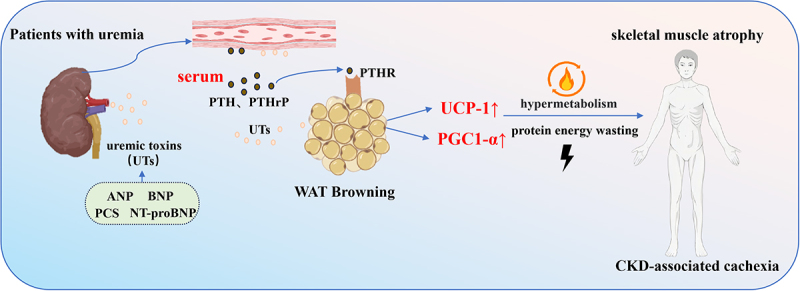
The browning of adipose tissue is linked to the advancement of kidney disease.

In CKD mouse models, WAT browning exacerbates cachexia [63,70]. This highlights the therapeutic potential of targeting WAT browning to alleviate CKD-associated cachexia. Serkan Kir et al. demonstrated that in 5/6 nephrectomies mice-a classic renal failure model, cachexia is associated with elevated circulating levels of parathyroid hormone (PTH). Secondary hyperparathyroidism is a common complication of CKD, particularly in patients undergoing dialysis [71]. This condition contributes to hypermetabolism and skeletal muscle atrophy, further exacerbating cachexia [72]. Hypermetabolism refers to a metabolic disorder characterized by a significant increase in resting energy expenditure (REE) of the body, serving as a hallmark pathological response in critical illnesses such as severe burns, cancer cachexia, sepsis, and mitochondrial diseases. Centred on metabolic imbalance, this state is manifested by elevated oxygen consumption and metabolic rate, uncontrolled catabolism of adipose tissue and skeletal muscle, and abnormal accumulation of circulating energy substrates, which in turn induce a series of adverse consequences including rapid weight loss, insulin resistance, and multiple organ dysfunction [73–75]. Mechanistically, elevated levels of circulating PTH and PTHrP activate the browning process through their shared receptor, parathyroid hormone receptor (PTHR) (Figure 4). Adipose tissue-specific knockout of the PTHR in CKD mice blocks browning, prevents adipose tissue depletion, and mitigates skeletal muscle atrophy [76].

Together, PAT communicates bidirectionally with the kidney, and its pathological browning contributes to disease progression in CKD. In clear cell renal cell carcinoma (ccRCC), parathyroid hormone-related protein (PTHrP) is also a key factor driving adipose tissue browning. To date, no studies have reported differences in circulating PTHrP concentrations between ccRCC and chronic kidney disease (CKD) models. Whether circulating PTHrP levels can serve as a biomarker for assessing disease progression remains an unanswered question warranting future investigation. Additionally, further research is needed to explore whether elevated lactate concentrations contribute to disease progression in CKD patients, analogous to its pathogenic role in ccRCC.

CKD cells-secreted uraemic toxins (UTs) increased the browning of WAT, increase protein energy expenditure, lead to skeletal muscle atrophy, and aggravate CKD-associated cachexia. elevated PTH/PTHrP binds to the PTH receptor (PTHR) on white adipose tissue (WAT), inducing WAT browning and upregulating thermogenic markers (UCP1, PGC1α). This ultimately increases energy expenditure and triggers a hypermetabolic state, accelerating CKD progression.

The Schematic model was created using BioRender (https://www.biorender.com/) and Adobe Illustrator.

## 5. Pathological adipose browning in burn

Burn injuries constitute one of the most severe forms of trauma, accounting for approximately 180,000 fatalities globally each year as a result of thermal injury [77]. The hypermetabolic response induced by burns contributes to extensive pathological damage across multiple tissues. During this hypermetabolic phase, the skin becomes significantly hypoxic. Chronic hypoxia further aggravates tissue damage through the accumulation of reactive oxygen species [78], which impairs wound healing, promotes immunosuppression, and increases the risk of infections, sepsis, and multiple organ failure [79]. Among the affected tissues, the liver, adipose tissue, and skeletal muscle display particularly pronounced metabolic adaptation [80,81]. Adipose tissue, in particular, serves a critical endocrine function following severe traumatic events such as burns [82]. Emerging evidence confirms that adipose tissue undergoes prominent thermogenic remodelling in response to burn injuries. Notably, classical BAT exhibits only transient upregulation of UCP1-mediated function, whereas WAT depots undergo consistent and sustained browning-a more robust and functionally relevant pathological change in the post-burn hypermetabolic state [83,84]. Mechanisms underlying burn-induced pathological browning implicate inflammatory cytokines, catecholamines, and lactate.

### 5.1. IL-6 is involved in the pathological browning of WAT in burns

The initial phase of a burn injury is marked by a pro-inflammatory response, known as systemic inflammatory response syndrome [85,86]. Elevated levels of inflammatory cytokines are a hallmark of burn patients, with interleukin-6 (IL-6) serving as a key mediator [87,88]. IL-6 is a multifunctional cytokine secreted by both metabolic tissues and immune cells in response to injury, and it is the only pro-inflammatory factor that remains consistently elevated systemically following burns [89]. In burn patients, peripheral blood IL-6 levels are significantly elevated [90]. Experimental animal models of third-degree burns (20% or 40% of total body surface area [TBSA] in rats) have demonstrated that serum IL-6 levels peak within the first hour after injury and are directly proportional to the size of the burn area [79].

After burn injury, IL-6 gene knockout (IL-6^−^/^−^) mice exhibited markedly attenuated WAT browning, with significantly reduced expression of key browning markers such as UCP1, which is associated with the loss of IL-6 signalling [91,92]. The exact mechanisms by which IL-6 promotes adipocyte browning remain incompletely understood. Studies in IL-6 gene knockout mice may have provided key insights. IL-6 knockout mice exhibited less severe fat atrophy and weight loss compared to wild-type mice subjected to similar burn injuries. Interestingly, transplantation of bone marrow from wild-type mice into IL-6 knockout mice restored burn-induced WAT browning and the associated metabolic changes, emphasizing the central role of IL-6 in mediating these processes [91].

### 5.2. Catecholamine-mediated browning is accompanied by lipolysis and FFA efflux after burns

In burn patients, catecholamine levels remain significantly elevated for years following the initial injury. This prolonged elevation is closely associated with stress, inflammation, hypermetabolism, and impaired immune function [93]. Catecholamines, primarily produced in the adrenal medulla, are released into the bloodstream in response to stress or injury [94]. Beyond their well-known roles in regulating metabolic and cardiac functions, catecholamines are critical mediators of adipose tissue browning. Catecholamines bind to β-3 adrenergic receptors on beige adipocytes, triggering a cascade of events that starts with the activation of adenylate cyclase. Substantial release of catecholamines occurs at sympathetic nerve terminals that innervate both BAT and WAT, promoting WAT browning and stimulating the expression of UCP1. This results in increased levels of cAMP, which in turn activates a canonical pathway leading to the upregulation of UCP1 [95]. In burns, this persistent catecholamine surge exacerbates the hypermetabolic response [96].

WAT browning-associated increased lipolysis facilitates FFA efflux, and the released FFAs stimulate macrophage polarization to create a self-sustaining cycle of WAT browning during hypermetabolic states [97,98]. In burn patients, this cycle leads to increased lipolysis and increased FFA release from adipose tissue. Burn-induced WAT browning and its associated increased lipolysis leads to hepatic steatosis, endoplasmic reticulum stress, and impaired hepatic fat oxidation [98].

### 5.3. Post-burn hyperlactatemia induces pathological browning

Although various cytokines have been recognized as activators of adipose tissue browning after burn injury, the contribution of metabolites in this process has received comparatively less attention. A recent study identified lactic acid-a central metabolic intermediate-as a direct inducer of WAT browning and a mediator of post-burn liver dysfunction, establishing its causal role in related signalling pathways [99]. As the main product of anaerobic respiration, lactate promotes browning in mouse white adipocytes by upregulating UCP1 expression.

Clinical evidence indicates that hyperlactatemia (blood lactate > 2 mmol/L) is common in burn patients and is accompanied by elevated expression of thermogenic markers in subcutaneous WAT (sWAT). Moreover, proteins involved in lactate uptake and conversion to pyruvate- such as monocarboxylate transporter 1 (MCT1) and lactate dehydrogenase (LDH)-are upregulated in these patients [100]. A positive correlation between MCT1 and UCP1 expression in WAT suggests that lactate uptake may initiate the browning process.

Similar results were observed in burned mice, where injections of sodium L-lactate led to increased circulating and intracellular lactate levels, worsened metabolic phenotypes, and significant weight loss [99]. After burn injury, lactate accumulates and is transported into adipocytes via MCT transporters. This elevates the intracellular NADH/NAD^+^ ratio and induces oxidative stress, subsequently upregulating fibroblast growth factor 21 (FGF21) and other batokines, thereby promoting WAT browning and thermogenesis [101]. This process elevates energy expenditure, contributing to post-burn hypermetabolism, weight loss, and liver dysfunction [99].

In contrast, treatment with phloretin, an MCT1 inhibitor, reduced lactate accumulation in WAT cells without leading to weight loss. It also attenuated browning and hepatic lipid infiltration in burned mice, accompanied by improved liver function [99]. These findings highlight the significant role of lactate-induced thermogenic browning in worsening hypermetabolism and tissue damage following burns.

**Figure 5.**
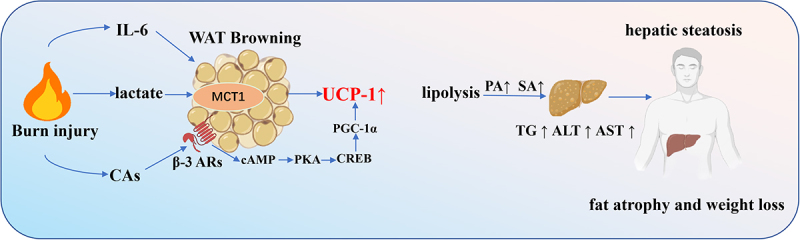
Browning of WAT induced by burn injury contributes to liver damage.

In summary, burn injury promotes WAT browning through inflammatory activation, lactate accumulation, and catecholamine signalling, collectively driving fat atrophy, weight loss, and hepatic injury. However, the detailed mechanisms through which lactate and IL-6 mediate WAT browning warrant further investigation (Figure 5).

Burn injury upregulates the inflammatory factor IL-6, which promotes WAT browning, increases energy expenditure, and leads to fat atrophy and weight loss. Burn-induced WAT browning and its associated increased lipolysis, elevating plasma levels of palmitic acid (PA) and stearic acid (SA), which induce hepatic endoplasmic reticulum stress, impair lipid oxidation, and ultimately cause hepatic steatosis. Concurrently, burn injury significantly elevates catecholamine levels, further stimulating WAT browning. Lipolysis-driven lipid breakdown increases free fatty acid (FFA) release, while lactate-mediated browning contributes to hepatic lipid infiltration and deposition. This leads to elevated triglycerides (TG) and FFAs, along with significantly increased circulating levels of alanine aminotransferase (ALT) and aspartate aminotransferase (AST)-markers of liver injury-thereby exacerbating hepatic steatosis and dysfunction.

The Schematic model was created using BioRender (https://www.biorender.com/) and Adobe Illustrator.

## 6. The lipolysis of beige fat accelerates the growth of atherosclerotic plaques

Atherosclerosis-associated cardiovascular disease (CVD) remains one of the leading causes of mortality and loss of productive life years globally [102,103]. Cold exposure has been identified as a significant environmental factor contributing to cardiovascular risk. Epidemiological studies reveal a positive correlation between decreased ambient temperature and increased cardiovascular disease-related mortality [104].

Cold exposure activates the sympathetic nervous system (SNS), leading to the release of epinephrine, which upregulates UCP1 expression, enhances thermogenesis, and activates BAT [105]. Notably, cold-induced browning also modulates the secretion of adipokines-among which adiponectin (a key anti-inflammatory, insulin-sensitizing adipokine) plays a critical regulatory role. Normally, adiponectin inhibits WAT browning by suppressing β3-adrenergic receptor signalling and reduces insulin resistance by promoting fatty acid oxidation in the liver and muscle [106,107]. However, cold exposure downregulates adiponectin expression in beige fat: this not only disinhibits browning to exacerbate lipolysis but also impairs insulin sensitivity-creating a ‘double hit’ that promotes hyperlipidaemia and vascular inflammation [108], two core drivers of atherosclerosis.

**Figure 6.**
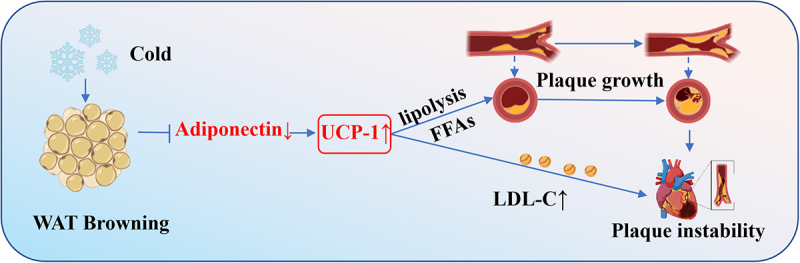
The lipolysis of beige fat accelerates the growth of atherosclerotic plaques.

In both ApoE^−^/^−^ and Ldlr^−^/^−^ mice, cold-induced UCP1 activation increases lipolysis, elevates plasma free fatty acid levels, enhances hepatic cholesterol synthesis, and raises the levels of small low-density lipoprotein (LDL) remnants-factors that collectively promote atherosclerotic plaque growth. Additionally, browning increases lipid deposition and inflammatory cell infiltration in plaques, reduces smooth muscle cells and collagen components, enlarges the necrotic core, and thins the fibrous cap, thereby enhancing plaque instability [109] (Figure 6). Interestingly, studies using ApoE^−^/^−^ UCP1 knockout mice have demonstrated protection from cold-induced plaque development [110]. Knockout of UCP1 completely blocks the lipolysis, hypercholesterolemia, and plaque lesions associated with cold-induced adipose tissue browning [109].

Given the factors driving plaque growth and instability, several therapeutic strategies have been proposed to mitigate cold- induced BAT activation and lipolysis. These include adiponectin-targeted interventions (e.g. adiponectin receptor agonists like AdipoRon or exogenous adiponectin supplementation, which restore adiponectin signalling to inhibit browning and improve insulin sensitivity) [111], simvastatin, ASIMOS (a β3-adrenergic antagonist), and preventive measures against cold exposure. Such interventions may help prevent the growth and destabilization of atherosclerotic plaques [109].

Under cold stimulation, the browning of WAT increases the level of small and medium low-density lipoprotein (LDL) promoting the growth of atherosclerotic plaques. The Schematic model was created using BioRender (https://www.biorender.com/) and Adobe Illustrator.

## 7. Pathological adipose browning in SARS-CoV-2 infection

The global SARS-CoV-2 pandemic has spurred extensive research into its pathogenesis and associated factors. Recent studies suggest a potential connection between adipose tissue browning and SARS-CoV-2 infection, highlighting its implications for disease progression [112,113].

SARS-CoV-2-infected patients often exhibit adipose tissue browning. Xu Jing et al. reported pronounced browning in both visceral and subcutaneous fat in SARS-CoV-2-infected animals, accompanied by significant weight loss within just four days post-infection. These observations were corroborated by post-mortem examinations of adipose tissue from COVID-19 fatalities [112]. In hamsters, COVID-19-induced adipose browning was associated with diffuse alveolar damage, hyaline membrane formation, pulmonary inflammation, and febrile responses-pathological features consistent with severe human COVID-19 pneumonia. Notably, patients exhibiting adipose browning tend to have poorer clinical outcomes, including elevated inflammatory markers and worsened respiratory function [112,114].

SARS-CoV-2 can directly infect mature adipocytes and a subset of adipose tissue macrophages. Viral RNA and mononuclear inflammatory infiltrates have been detected in the adipose tissue of deceased COVID-19 patients. Although angiotensin-converting enzyme 2 (ACE2) expression is relatively low in adipocytes and macrophages, the virus successfully enters these cells, possibly through alternative receptor mechanisms [115]. Cantuti-Castelvetri et al. identified neuropilin-1 (NRP1) as a co-receptor that facilitates viral entry by interacting with furin-cleaved substrates [116]. While infected macrophages do not produce infectious virions, they amplify viral RNA and synthesize viral proteins, thereby stimulating stromal vascular cells (SVCs) to initiate inflammatory responses. This process promotes the secretion of various cytokines and chemokines, such as interferon-γ-induced protein-10 (IP-10), platelet-derived growth factors (PDGF-AA, PDGF-AB/BB), IL-4, macrophage migration inhibitory factor (MIF), vascular endothelial growth factor A (VEGFA), and macrophage colony-stimulating factor 1 (MCSF1), activating signalling pathways that drive WAT browning [115].

SARS-CoV-2 infection also induces significant hypoxia in lung tissue, upregulating HIF1α and carbonic anhydrase 9 (CA9). This hypoxic state extends to subcutaneous adipose tissue, increasing the expression and circulating levels of VEGF-a key target of HIF1α. Known for its roles in angiogenesis and vascular permeability, VEGF elevates microvascular density in subcutaneous WAT [117–119]. Moreover, VEGF promotes a browning phenotype by modulating vascular-adipocyte interactions, leading to increased energy expenditure and weight loss [120,121].

**Figure 7.**
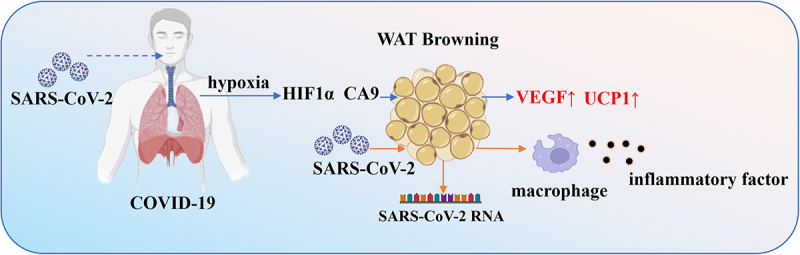
SARS-CoV-2 infection is accompanied by adipose tissue browning.

In summary, adipose tissue browning is associated with SARS-CoV-2 infection. The underlying mechanisms may involve direct viral infection of adipocytes, macrophage-derived inflammatory factors, as well as pulmonary hypoxia-mediated pathways involving HIF1α and CA9 (Figure 7).

The infected with SARS-CoV-2 increased expressions of hypoxia-inducing factors HIF1α and CA9 in lung, inducing brown of WAT. In addition, SARS-CoV-2 can directly infect adipocytes and macrophages, mediate inflammatory response and browning. The Schematic model was created using BioRender (https://www.biorender.com/) and Adobe Illustrator.

## 8. Pathological adipose browning in sepsis

Sepsis is a life-threatening systemic inflammatory response syndrome caused by dysregulated host reactions to infection, which is a major cause of mortality in intensive care units worldwide [122]. Accumulating evidence has identified pathological adipose browning as a crucial pathogenic mediator in sepsis progression, which not only disrupts systemic metabolic homoeostasis but also exacerbates inflammation and multiple organ damage, ultimately worsening patient prognosis [123]. Clinical and preclinical studies have confirmed the presence of significant adipose browning in sepsis, with distinct differences observed between obese and non-obese populations [124]. This process is regulated by multiple pathways such as the adrenergic receptor pathway, inflammatory factor network, hypoxia response, and natriuretic peptide signalling, forming a complex pathological regulatory loop [123].

A study established a polymicrobial sepsis model in C57BL/6 mice via caecal ligation and puncture (CLP)-the gold standard method for simulating clinical sepsis. The mice were divided into an obese group (fed a high-fat diet) and a non-obese group (fed a normal diet). Sepsis induced significant WAT browning in non-obese mice, as evidenced by marked upregulation of UCP1 expression, while obese mice showed strong resistance to sepsis-induced WAT browning with notably lower UCP1 expression than non-obese septic mice [125].

The NE, a non-selective α/β-adrenergic receptor agonist. NE further elevated UCP1 levels in non-obese mice but had no significant effect on obese mice. These results confirm the β-adrenergic receptor pathway mediates this difference: sepsis triggers a surge in catecholamine release, and NE binds to β-adrenergic receptors on WAT to increase intracellular cAMP levels, thereby promoting lipolysis and activating UCP1 to induce browning. Obese mice, however, have reduced expression of β-adrenergic receptor subtypes in adipose tissue and impaired function of HSL-making them unable to efficiently initiate the browning process even under NE stimulation [125].

Pathological adipose browning is closely correlated with the severity of sepsis. Non-obese septic mice with significant browning presented more severe clinical phenotypes, such as hypothermia, metabolic acidosis, elevated lung myeloperoxidase (MPO) activity (a hallmark of neutrophil infiltration), and increased liver injury markers. In contrast, obese mice without obvious browning maintained near-normal blood pH, reduced lung MPO activity, and alleviated liver damage. This phenotypic discrepancy is mediated by the inflammatory factor regulatory network: non-obese septic mice had substantially higher plasma levels of TNF-α and IL-6, while obese mice sustained stable inflammatory cytokine levels during sepsis-an important contributor to their resistance to browning. As a key sepsis-related inflammatory factor, IL-6 drives M2 macrophage polarization; M2 macrophages then locally secrete catecholamines to activate the β3-adrenergic receptor pathway in adipocytes, further amplifying adipose browning and systemic inflammation [123,125].

Some reviews summarized adipose browning in critical illnesses including sepsis, confirming that extensive WAT browning-affecting both subcutaneous and visceral fat depots-is a common pathological feature of sepsis. Activated BAT and Beige AT synergistically exacerbate systemic hypermetabolism, accelerating nutrient depletion and deteriorating metabolic imbalance. Beyond the core regulatory pathways of adrenergic receptors and inflammatory factors, two complementary mechanisms are involved: first, sepsis-induced tissue hypoxia upregulates the expression of HIF and VEGF in adipose tissue, where HIF enhances microvascularization and VEGF promotes NE release via sympathetic nervous system activation, collectively inducing WAT browning; second, sepsis-associated cardiac dysfunction leads to the accumulation of ANP and BNP, which activate cAMP in WAT, phosphorylate hormone-sensitive lipase and perilipin, induce lipid droplet breakdown, and provide substrates for mitochondrial respiration to facilitate adipose browning [123,126].

**Figure 8.**
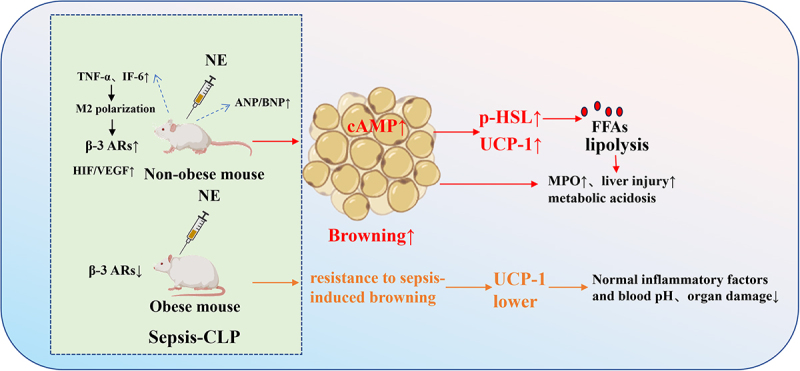
Pathological regulation of adipose tissue browning in sepsis and obesity-associated differences.

Current research on pathological adipose browning in sepsis has laid a solid foundation for elucidating its pathogenic mechanisms (Figure 8) but still has notable limitations: evidence is predominantly derived from animal models, with insufficient clinical data to validate the correlation between adipose browning markers (e.g. UCP1) and sepsis severity or prognosis; the crosstalk between different regulatory pathways remains unclear; and clinically translatable targeted therapeutic strategies are lacking. Therapeutically, targeting key nodes in the browning pathway-such as β-blockers for inhibiting the adrenergic pathway, IL-6 inhibitors for mitigating inflammation-driven browning, or haemodialysis for clearing excess natriuretic peptides-may offer novel approaches to alleviate sepsis-related hypermetabolism and organ damage. Future studies should focus on clinical cohort research and translational investigations to confirm the potential of adipose browning as a prognostic marker and therapeutic target in sepsis.

In the CLP-induced sepsis model, pathological browning of WAT in non-obese mice is triggered via multiple pathways: sepsis-induced inflammatory factors (TNF-α, IL-6) drive M2 macrophage polarization, and the catecholamines secreted by M2 macrophages activate β-3ARs on adipocytes. Meanwhile, sepsis-associated hypoxia upregulates HIF/VEGF expression, which further promotes NE release. Ultimately, the cAMP signalling pathway activates p-HSL and UCP1, triggering lipolysis and browning. This process is accompanied by increased release of FFAs, exacerbating elevated lung MPO activity, liver injury, and metabolic acidosis, thus worsening sepsis severity. In contrast, obese mice exhibit resistance to sepsis- and NE-induced browning due to reduced β-3ARs expression in adipose tissue: UCP1 levels remain low, inflammatory factor levels and blood pH are more stable, and organ damage is significantly alleviated.

## 9. Mechanistic integration

Although the diseases discussed in this review-ranging from breast cancer and kidney diseases to severe burns and COVID-19 infection-differ significantly in their aetiology and pathological contexts, a striking commonality is their association with pathological browning of WAT. By integrating the mechanisms driving browning across these diverse disease models, we can move beyond isolated observational phenomena to extract a common molecular framework that transcends individual diseases. This framework reveals that pathological browning does not occur randomly but is mediated by a limited set of conserved signalling pathways and triggering factors.

First, multiple diseases share upstream triggers of browning. Inflammatory signals (such as the role of IL-6 in burns and CKD), hypoxia and its core mediator HIF1α (in breast cancer, COVID-19, and burns), as well as specific metabolites (such as lactate in ccRCC and burns), collectively form a common ‘pathological stimulus pool’. These factors are produced in large quantities within the unique microenvironments of their respective diseases, serving as the initial signals that initiate adipocyte reprogramming.

Secondly, these diverse triggering factors converge onto a limited number of highly conserved downstream signalling hubs. Among these, the PKA pathway serves as a critical junction: it can be activated both by G protein-coupled receptor (GPCR) ligands (such as ADM in breast cancer, PTHrP in ccRCC, and PTH and ANP in CKD and by β-adrenergic receptors (e.g. catecholamines in burns). The activation of PKA acts as a powerful universal switch, directly upregulating UCP1 expression and driving lipolysis.

Ultimately, these distinct pathways lead to a common terminal effect: the browning of adipose tissue. This process is characterized by enhanced UCP1-mediated thermogenesis and increased lipolysis. This directly results in two consequences with systemic implications: significant energy expenditure, exacerbating cancer- and CKD-related cachexia and muscle atrophy; and substantial release of lipolytic products (FFAs) and metabolic byproducts (such as lactate). These molecules enter the circulatory system, fuelling tumour cell growth (in breast cancer and ccRCC), accumulating in the liver to cause steatosis and dysfunction (in burns), or promoting the progression of atherosclerotic plaques (under cold stimulation).

Thus, pathological adipose browning can be conceptualized as a self-sustaining vicious cycle: the primary disease produces specific pathological stimuli; these stimuli activate conserved signalling hubs (e.g. PKA, HIF1α); leading to adipose tissue browning; the released metabolites (FFAs, lactate) and consumed energy in turn exacerbate the progression of the primary disease and systemic damage. This integrative perspective suggests that therapeutic strategies targeting these common pathways (e.g. PKA, HIF1α) or effector products (e.g. lactate) may hold broad-spectrum therapeutic potential across multiple diseases, providing a theoretical foundation and direction for the future development of novel therapies against pathological browning and its detrimental consequences.

## 10. Conclusion and prospect

**Figure 9.**
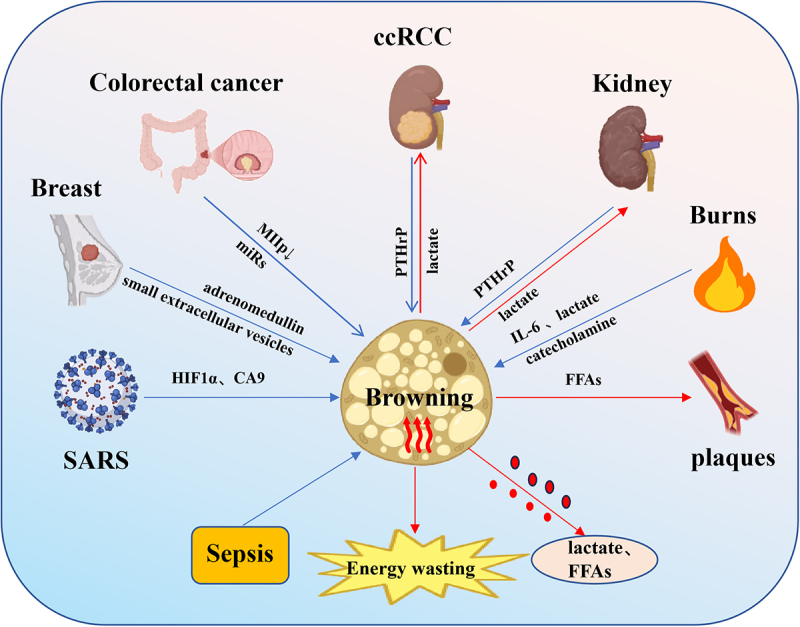
Pathological adipose browning.

Numerous studies have indicated that adipose tissue browning can ameliorate disease conditions [127,128]. However, this review consolidates substantial compelling evidence that expands the concept of adipose browning beyond its conventional metabolic benefits paradigm. Instead, this review synthesizes that WAT browning is a common pathological feature accompanying the progression of diverse conditions, including breast cancer, renal cell carcinoma, chronic kidney disease, burn injury, and infections (Figure 9). It further reveals that this browning, when accompanying disease-specific microenvironments, can exacerbate disease progression.

In different disease scenarios, the upregulation of beige fat in adipose tissue highlights the elusive plasticity of adipose tissue. We refer to this phenomenon as ‘disease-associated’ or ‘pathological browning’, which likely represents an adaptive or stress-induced response to specific pathological stimuli, such as inflammation, cytokines, and metabolic waste products. Current evidence does not uniformly support an exclusively beneficial or detrimental role; rather, it depicts a more complex picture wherein the functional consequences of browning may vary depending on the disease type, stage, and microenvironment- demonstrating context-dependent effects.

Potential interventions targeting pathological adipose browning exist for diseases like kidney disease, burns, and COVID-19. Including growth hormone/vitamin D/anakinra (CKD), TKI-PKA inhibitor combinations (ccRCC), lytic cocktails/propranolol (burns), and VEGF blockers (COVID-19). But they carry critical limitations: preclinical-only validation and no clinical safety/efficacy data. Future therapies should prioritize precise pathological browning modulation: clarify disease-specific drivers, distinguish beneficial vs. pathological browning, develop targeted tools, use biomarkers (e.g. UCP1, AZGP1, HOXC10) for patient stratification, and optimize doses/safety to turn browning modulation into safe, precise treatments.

Future research must transition from merely observing these associations to definitively elucidating the underlying mechanistic drivers and causal relationships. Firstly, identifying the precise pathological triggers (e.g. tumour-derived factors, uraemic metabolites, inflammatory mediators) and downstream signalling pathways that initiate browning in disease states, while distinguishing these from pathways activated by cold exposure or β-adrenergic stimulation. Secondly, determining whether pathological browning functions as a bystander phenomenon, a compensatory response, or an active contributor to disease pathogenesis. Ultimately, deciphering the duality of adipose browning-both its protective effects and potential pathological roles-is crucial for a comprehensive understanding of inter-organ communication in disease and for developing targeted interventions that modulate this process to achieve therapeutic benefits.

In certain specific diseases, including breast cancer, colorectal Cancer, renal cell carcinoma, chronic kidney disease, burn injury, infections and sepsis WAT browning is consistently observed. Furthermore, browning of perirenal adipose tissue has been shown to exacerbate the progression of renal cancer, while increased release of free fatty acids from beige adipose in cold environments promotes the growth of atherosclerotic plaques.

The Schematic model was created using BioRender (https://www.biorender.com/) and Adobe Illustrator.

## Data Availability

Data sharing is not applicable to this article as no data were created or analysed in this study.
